# Radiological Manifestations of Ketamine Abuse

**DOI:** 10.5334/jbsr.2537

**Published:** 2021-07-08

**Authors:** Amin Da’meh, Alexandre Al-Awa, Sadeq Da’meh

**Affiliations:** 1 VUB, BE; 2 UZ Brussel, BE; 3 The Jordanian Royal Medical Services, JRMS, Jordan

**Keywords:** Ketamine, abuse, pneumomediastinum, computed tomography, cystitis, hydronephrosis

## Abstract

**Teaching point:** Awareness of the radiological manifestations helps recognition of ketamine abuse.

## Case presentation

A 26-year-old male patient presented to the emergency department complaining of abdominal pain, dysuria frequency, and hematuria. The patient was known to be an abuser of nasal Ketamine for four years. Laboratory results showed a high level of serum creatinine (3.05 mg/dl), disturbed liver tests, high C-Reactive Protein (126.7 mg/l), and macroscopic hematuria (1735 p/µl).

**Figure 1:**
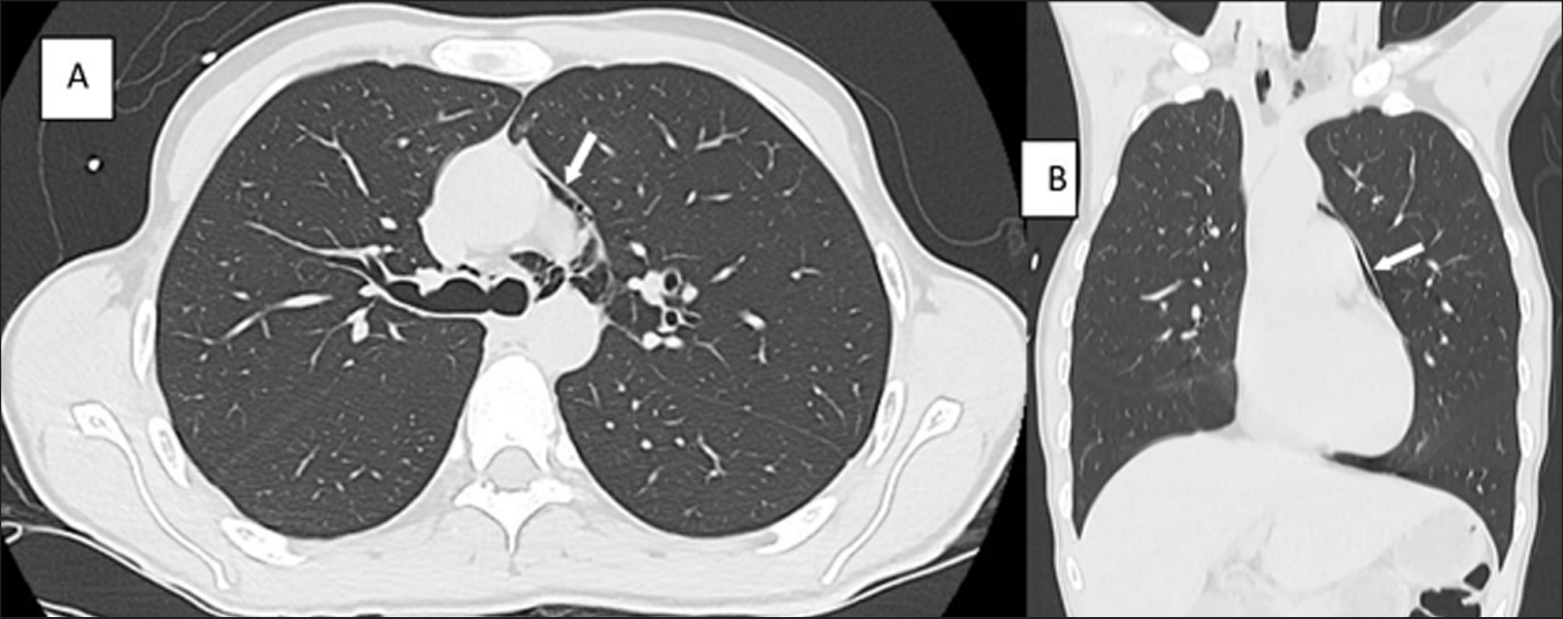


**Figure 2:**
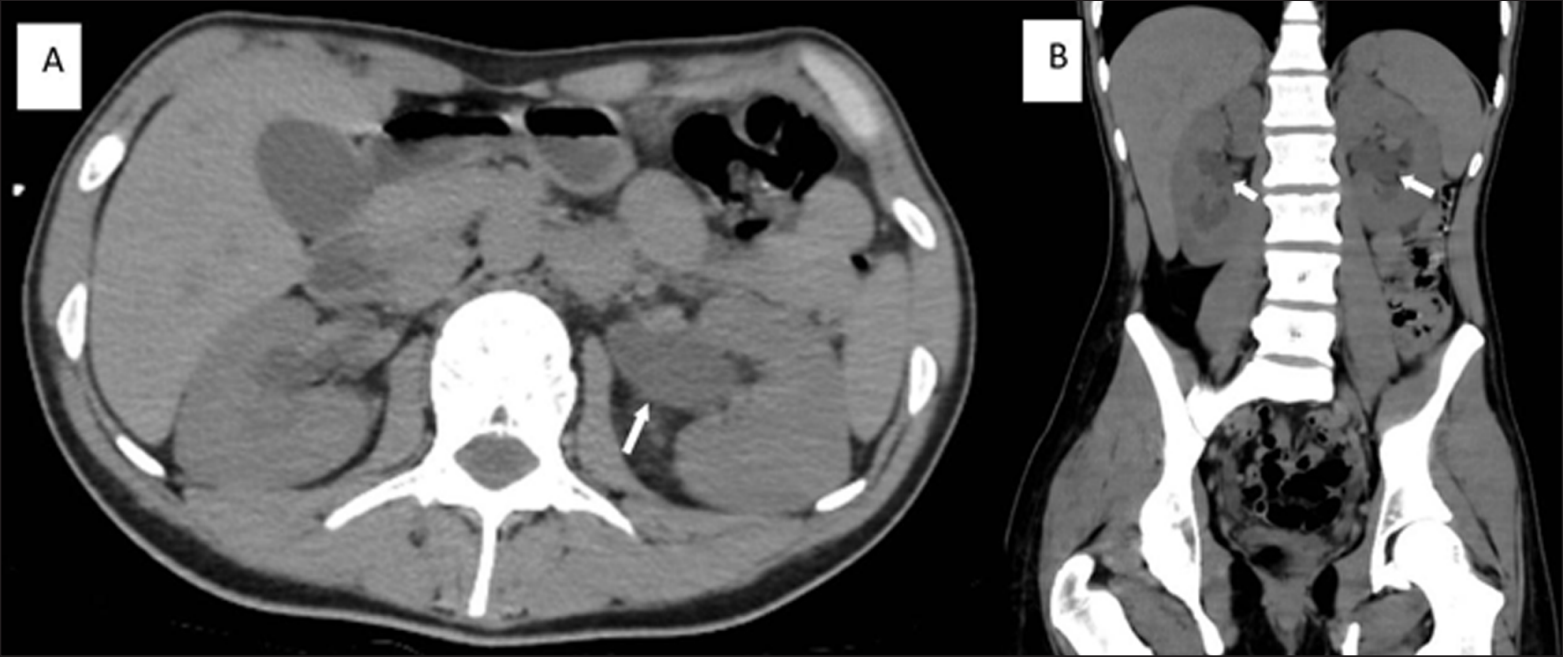


**Figure 3:**
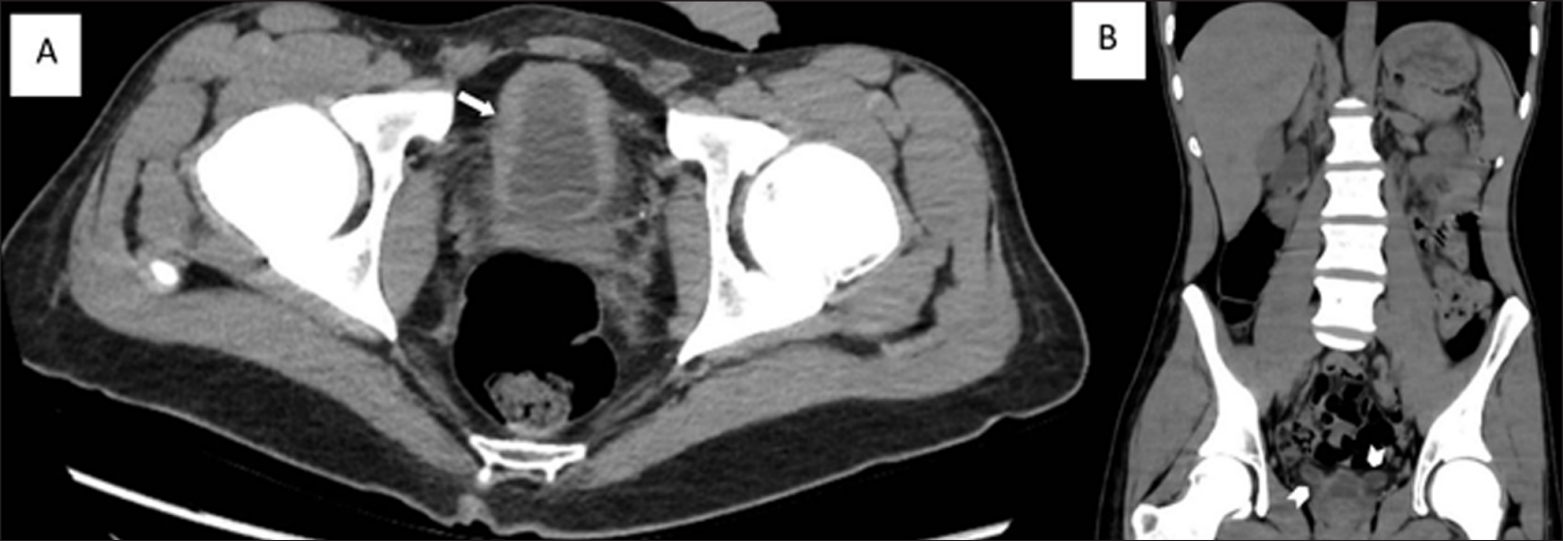


A non-enhanced thoracoabdominal CT showed free air in the mediastinum (***Figure 1A*** and ***1B***, arrows), bilateral hydronephrosis (***Figure 2A*** and ***2B***, arrows), a small bladder with irregular thickened walls surrounded by fat infiltration (***Figure 3A***, arrow), and a marked dilatation of the distal ureters (***Figure 3B***, arrowheads).

## Discussion

Ketamine is a short-acting anesthetic agent commonly used for intubation, procedural sedation and short-term treatment of inflammatory bowel disease. It acts as a non-competitive antagonist at N-methyl-D-Aspartate (NMDA) receptors. This action may account for dissociation feelings, development of hallucinations, floating sensations, near-death experience, and therefore recreational ketamine abuse has been increasingly reported for many years among young adults. Ketamine may be snorted, ingested, smoked, or injected.

Ketamine abuse commonly leads to urinary tract destruction “The Ketamine bladder syndrome,” which is thought to be caused by the smooth muscle relaxing property of ketamine. Chronic Ketamine abuse damages the urinary bladder, causing ulcers, cystitis, and fibrosis, resulting in urinary incontinence, hematuria, bladder overactivity, and shrinkage, and in the later stage, uretero-hydronephrosis [1]. It also may cause epigastric pain, gastritis, and biliary system abnormalities, that is, irregularity and non-obstructive biliary dilatation associated with impairment of the liver function.

Lastly, occurrence of pneumomediastinum, subcutaneous emphysema, and pneumorrhachis is thought to be caused by barotrauma due to the sudden increase in the interalveolar pressure following passive apnea and/or cough during the insufflations of Ketamine. After alveolar overdistention and rupture, air diffuses into the interstitial space, reaching the hilum and leading to a pneumomediastinum. It may then track along the fascial layers of the neck and the chest wall, causing subcutaneous emphysema, and eventually migrates through the intervertebral foramens to the epidural space causing pneumorrhachis.

## References

[1] Williams J, Hsu E, Flamer-Caldera A, et al. The Special K constellation, a rare presentation of Ketamine use: A case report. Cureus. 2019; 11(5): e4766. DOI: 10.7759/cureus.4766 31363447 PMC6663056

